# The synapsis checkpoint and the LIN-35/DREAM complex promote temperature stress-induced increases in germline apoptosis in *Caenorhabditis elegans*

**DOI:** 10.1093/g3journal/jkaf228

**Published:** 2025-09-26

**Authors:** Frances V Compere, Kristen A Quaglia, Margaret N Crespo Cruz, Hannah N Lorenzen, Samantha H Oswald, Katherine Uttal, Lisa N Petrella

**Affiliations:** Department of Biological Sciences, Marquette University, Milwaukee, WI 53217, United States; Department of Biological Sciences, Marquette University, Milwaukee, WI 53217, United States; Department of Biological Sciences, Marquette University, Milwaukee, WI 53217, United States; Department of Biological Sciences, Marquette University, Milwaukee, WI 53217, United States; Department of Biological Sciences, Marquette University, Milwaukee, WI 53217, United States; Department of Biological Sciences, Marquette University, Milwaukee, WI 53217, United States; Department of Biological Sciences, Marquette University, Milwaukee, WI 53217, United States

**Keywords:** LIN-35, CED-9, DREAM, synapsis checkpoint, temperature stress, germline apoptosis, WormBase

## Abstract

As modest increases in temperature become more common due to global climate change, organisms are being subjected to moderate temperature stresses that can disproportionally affect fertility. Species that can buffer fluctuations in temperature through tissue or cellular responses in the germ line will therefore be more likely to survive moderate temperature stresses. Currently, what mechanisms are used in the germ line to facilitate maintenance of fertility under moderate temperature stress remain unknown. To address this, we investigated how germline apoptosis is modulated in *Caenorhabditis elegans* nematodes in response to moderate temperature stress. We found that wild-type animals increase their germline apoptosis levels from the physiological baseline in response to the moderate temperature stress. This induction of germline apoptosis was dependent on known and novel regulators of germline apoptosis including members of the conserved DREAM (Dp, Retinoblastoma (Rb)-like, E2F, MuvB) complex: LIN-35/pRB, LIN-54, and LIN-37, and proteins that regulate the synapsis checkpoint, BUB-3 and PCH-2. Additionally, repression of CED-9 function, the *C. elegans* Bcl2 ortholog, was necessary for full induction of apoptosis during moderate temperature stress. Finally, we found that changes in cytoplasmic streaming are correlated with changes to oocyte provisioning in wild-type animals but not mutants. Together, these data suggest an expanded role for LIN-35, the MuvB core of the DREAM complex, CED-9, and the synapsis checkpoint in maintaining fertility by activating apoptosis during moderate temperature stress.

## Introduction

For a species to maintain population size, organisms must respond to environmental stressors to maintain fertility. Given the unique contributions of oocytes to both the genome and cytoplasm of embryos, the oogenic germline plays a major role in ensuring fertility and progeny fitness under stressful conditions. Apoptosis has been shown to be a mechanism used in the oogenic germ line to respond to a number of different stressors in *C. elegans* including DNA damage, osmotic stress, oxidative stress, starvation, ethanol stress, aging, and different types of elevated temperature stress (Gartner et al. 2000; Salinas et al. 2006; Andux and Ellis 2008; Poullet et al. 2015; Fausett et al. 2021). Moderate increases in temperature, well below the range that stimulate a heat shock response, are known to negatively affect fertility in a wide range of organisms including mammals, plants, insects, and nematodes (Zinn et al. 2010; Takahashi 2012; Petrella 2014; Gandara and Drummond-Barbosa 2023; Tushabe et al. 2023). Given the projected continuing increases in temperatures with global climate change, we are interested in the cellular mechanisms that contribute to apoptosis during moderate temperature stress, and whether this increase in apoptosis could play a role in preservation of oocyte quality.

In *C. elegans* hermaphrodites, the germ line in adults is oogenic and the sole site of postdevelopmental apoptosis (Gartner et al. 2008). Each hermaphrodite gonad is a U-shaped tube that contains germ cells surrounded by a thin layer of supporting somatic cells (Hubbard and Greenstein 2005). In the section of the germ line distal to the uterus, the germline nuclei are only partially surrounded by a cell membrane and thus are in a syncytium with a shared core of cytoplasm called the rachis. By convention, these nuclei are termed germ cells despite being only partially cellularized (Raiders et al. 2018). Germ cells initially undergo mitosis and then transition into meiosis as they move down the gonad. During nonstressful conditions, ∼50% of germ cells undergo what is termed physiological apoptosis as they approach the bend of the gonad (Gumienny et al. 1999). These dead germ cells are then engulfed by the surrounding somatic sheath cells and most of the associated cytoplasm and organelles are directed to the remaining living nuclei through cytoplasmic streaming (Ellis et al. 1991; Wolke et al. 2007). Cytoplasmic streaming is a mechanism by which the nuclei destined to become mature oocytes obtain a large store of cytoplasm/organelles as they cellularize (Wolke et al. 2007).

Various stress conditions trigger an increase in apoptosis above the level of physiological apoptosis. Thus, apoptosis can act as a cellular response to environmental stress in the oogenic germ line resulting in 2 related outcomes that may help to maintain fertility (Gartner et al. 2008 ; Cao and Pocock 2022). First, the removal of damaged nuclei limits the inheritance of damaged genomes, and second, an increase in the amount of cytoplasm entering the remaining oocytes could provide increased resources for early embryonic health. Like other stressors, moderate temperature stress of 26 to 27 °C in *C. elegans* has also been shown to increase the level of germline apoptosis (Poullet et al. 2015). At this temperature range, *C. elegans* goes from having few progeny (26 °C) to being basically sterile (27 °C) (Petrella 2014). While the molecular mechanisms leading to increased levels of apoptosis are well understood for other stressors, the molecular mechanisms leading to increased apoptosis under moderate temperature stress are unknown.

**Fig. 1.**
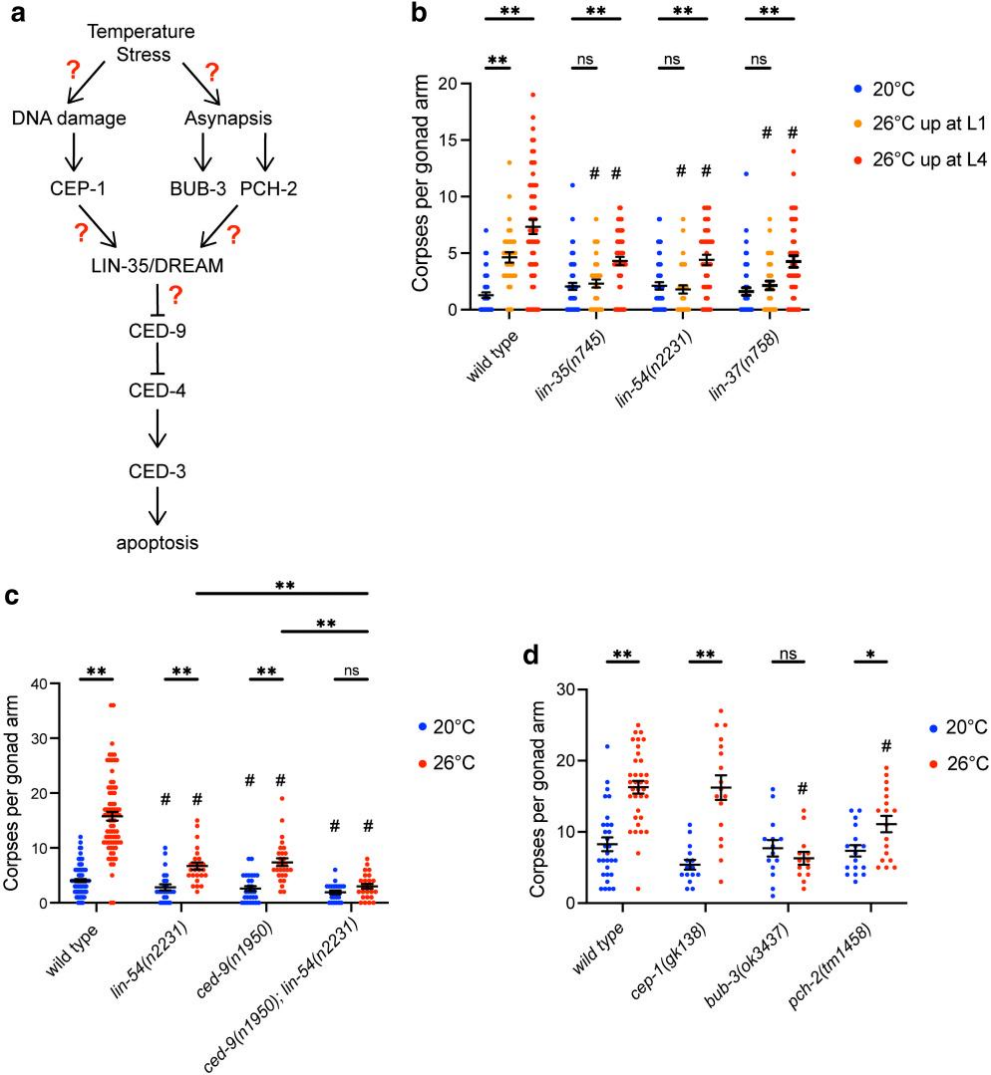
LIN-35/DREAM complex and the synapsis checkpoint are necessary for apoptosis induction during temperature stress. a) Model of aspects of the apoptosis pathway being tested. Where moderate temperature stress could work by activation of the DNA damage checkpoint (through CEP-1) or synapsis checkpoint (through BUB-3 and PCH-2) to activate LIN-35 and the DREAM complex. LIN-35/DREAM complex would then activate germline apoptosis through suppression of CED-9 function or expression, leading to CED-4 activation of CED-3 resulting in increased apoptosis. In all experiments, germ cell corpses were counted per gonad arm using *ced-1::gfp* in hermaphrodites 24 h post-L4 stage in the indicated genetic backgrounds. Hermaphrodites were either maintained continually at 20 °C (blue), upshifted to 26 °C at the L1 stage (orange), or upshifted to 26 °C at the L4 stage (red) with each dot representing an individual gonad. b) Germline apoptosis induction during temperature stress is weaker or gone in *lin-35(n745)* and DREAM complex Muv B core mutants. *n* = 34 to 58 gonads. c) Germline apoptosis induction during temperature stress is weaker in *ced-9(n1950)* mutants and gone in *ced-9(n1950); lin-54(n2231)* double mutants. *n* = 24 to 77 gonads. d) Germline apoptosis induction during temperature stress is gone in *bub-3(ok3437)* or weaker in *pch-2(tm1458)* mutants, but is at wild-type levels in *cep-1(gk138)* mutants. *n* = 13 to 35 gonads. *P ≤ 0.05, **P ≤ 0.01 significantly different within the genotype compared with 20 °C or (c only) compared between mutants at 26 °C, #*P* ≤ 0.05 significantly different than wild type at the same temperature, using a nonparametric pairwise Wilcoxon *t*-tests with Benjamani and Hochberg correction for multiplicity. Error bars indicate ±SEM.

Germline apoptosis in *C. elegans* uses the same conserved signaling pathway found across eukaryotes (Fig. 1a) (Ellis and Horvitz 1986; Gartner et al. 2008). CED-9, the *C. elegans* anti-apoptotic BCL2 homolog, functions to repress activation of the core apoptotic caspase machinery by sequestering CED-4, the *C. elegans* Apaf homolog (Hengartner et al. 1992; Spector et al. 1997; Chen et al. 2000). CED-9 sequestration of CED-4 can be inhibited by interactions with pro-apoptotic BCL2 proteins such as EGL-1 and/or CED-13, but only during stress-induced germline apoptosis (Gumienny et al. 1999; Schumacher et al. 2005; Ye et al. 2014). When CED-9 protein function is inhibited, CED-4 is released and goes on to activate CED-3, the terminal caspase that activates apoptosis (Chinnaiyan et al. 1997; Spector et al. 1997; Chen et al. 2000). LIN-35, the sole *C. elegans* homolog of the mammalian pocket proteins, has been shown to be necessary for the increase in germline apoptosis under starvation and DNA damage conditions (Schertel and Conradt 2007; Láscarez-Lagunas et al. 2014). In both stress-induced apoptosis and physiological apoptosis, LIN-35 has been shown to modulate apoptosis levels through repression of *ced-9* mRNA expression (Schertel and Conradt 2007; Láscarez-Lagunas et al. 2014). However, LIN-35 does not have DNA-binding activity; therefore, its repressive activity is mediated through its interactions within protein complexes. The most well-studied LIN-35 interaction is with the conserved DREAM complex (Dp, Retinoblastoma (Rb)-like, E2F, MuvB) (Harrison et al. 2006; Goetsch et al. 2017). The DREAM complex is made up of an E2F/DP dimer linked through a pocket protein, such as LIN-35, with the Muv B core (Harrison et al. 2006; Goetsch et al. 2017). The Muv B core in *C. elegans* is made up of 5 proteins, including LIN-54 and LIN-37. No studies to date have looked at a role for the Muv B core of the DREAM complex in regulation of *C. elegans* germline apoptosis. However, the conserved members of the DREAM complex, including LIN-35, have been shown in embryos to bind to the *ced-9* operon (Goetsch et al. 2017). Therefore, members of the Muv B core of the DREAM complex are potential cofactors for LIN-35 in induction of increased germline apoptosis during stress, including moderate temperature stress.

Here, we investigate the mechanisms leading to increased apoptosis during moderate temperature stress and explore the effects of moderate temperature stress on cytoplasmic streaming, oocyte size, and ovulation rate. We find that moderate temperature stress-induced apoptosis depends on both LIN-35 and the Muv B core in addition to repression of CED-9 function. We also find that moderate temperature stress-induced apoptosis requires the synapsis checkpoint, but not the DNA damage checkpoint. During moderate temperature stress, there is also an increase in cytoplasmic streaming in wild type with a concomitant increase in oocyte size. Overall, these findings further underscore LIN-35 and the Muv B core of the DREAM complex as general regulators of stress-induced germline apoptosis. In turn, increased apoptosis during moderate temperature stress provides a mechanism for increased oocyte size that may be protective for embryonic development.

## Methods

### Strains and nematode culture

*C. elegans* were cultured under standard conditions (Brenner 1974) on NGM plates seeded with *Escherichia coli* strain AMA1004 at 20 °C unless otherwise noted. N2 was used as the wild-type control unless otherwise noted. Strains used in this study were N2, MT8841  *lin-54(n2231) IV,*  MT10430  *lin-35(n745) I,*  MT5470  *lin-37(n758) III*, MT4770  *ced-9(n1950) III,*  MD701  *bcls39(lim-7p::ced-1::GFP) V,* LNP0089 *lin-35(n745); bcls39(lim-7p::ced-1::GFP) V,* LNP0091 *lin-54(n2231) IV; bcls39(lim-7p::ced-1::GFP) V,* LNP0092 *lin-37(n758) III; bcls39(lim-7p::ced-1::GFP) V*, LNP0266 *ced-9(n1950) III; bcls39(lim-7p::ced-1::GFP) V,* LNP0268 *ced-9(n1950) III; lin-54(n2231) IV*; *bcls39(lim-7p::ced-1::GFP) V*, LNP0012 *bub-3(ok3437) II: bcIs39(lim-7p::ced-1::GFP) V*, LNP0127 *pch-2(tm1458) II; bcIs39(lim-7p::ced-1::GFP) V,* JBC1 *cep-1(gk138) I; bcIs39(lim-7p::ced-1::GFP) V*. All LNP strains were made for this study. The N2 strain was from the laboratory of Susan Strome. The JBC1 strain was a gift from the laboratory of Dr. Jill Bargonetti (Hoffman et al. 2014). All other strains were provided by the CGC, which is funded by NIH Office of Research Infrastructure Programs (P40 OD010440).

### CED-1::GFP apoptosis assay

#### Treatments

*Control for temperature.* Worms were grown to the L4 stage at 20 °C, then isolated and maintained at 20 °C for 24 h prior to imaging. For Fig. 1b, 3 to 4 biological replicates were performed, with 5 to 10 worms analyzed per replicate for a total of *n* = 50–54 germline arms per genotype. For Fig. 1c, 5 to 11 biological replicates were performed, with 3 to 5 worms analyzed per replicate for a total of *n* = 24 to 77 germline arms per genotype. For Fig. 1d, 3 to 7 biological replicates were performed, with 2 to 8 worms analyzed per replicate for a total of *n* = 14 to 29 germline arms per genotype.

*Upshift to 26 °C at L1 stage.* Worms were grown to the L4 stage at 20 °C, then 10 to 20 P0 L4 worms were isolated and maintained at 20 °C for 24 h until they all reached the adult stage. All P0 worms were moved to new plates and allowed to lay embryos for 3 h before being removed. F1 embryos were allowed to hatch and grow for 24 h at 20 °C, then upshifted to 26 °C until the L4 stage. F1 L4 worms were then isolated and maintained at 26 °C for 24 h prior to imaging. For Fig. 1b, 2 biological replicates were performed, with 8 to 10 worms analyzed per replicate for a total of *n* = 34 to 38 germline arms per genotype.

*Upshift to* 26 °C *at L4 stage.* Worms were grown to the L4 stage at 20 °C, then isolated and upshifted to 26 °C for 24 h prior to imaging. For Fig. 1b, 3 biological replicates were performed, with 5 to 10 worms analyzed per replicate for a total of *n* = 40 to 58 germline arms per genotype. For Fig. 1c, 5 to 11 biological replicates were performed, with 3 to 5 worms analyzed per replicate for a total of *n* = 25 to 77 germline arms per genotype. For Fig. 1d, 2 to 6 biological replicates were performed, with 2 to 10 worms analyzed per replicate for a total of *n* = 13 to 35 germline arms per genotype.

#### Apoptosis scoring

Worms were mounted on a 2% agarose pad in 1 mM levamisole in 1X M9 buffer. For data in Fig. 1b, both germline arms were scored for GFP-positive apoptotic cells using the *ced-1::GFP* transgene on a Nikon Eclipse TE2000-S inverted microscope equipped with a Plan Apo 60X/1.25 numerical aperture oil objective. For Fig. 1c and d, images of *ced-1::GFP* were acquired using Leica Application Suite Advanced Fluorescence 3.2 software using Leica CTR6000 deconvolution inverted microscope with a Hamatsu Orca-R2 camera and Plan Apo 63x/1.4 numerical aperture oil objective. One germline per animal was imaged. Image stacks were analyzed in FIJI to count apoptotic cells (Schindelin et al. 2012).

#### Statistical analysis of apoptosis data

Prior to statistical analysis, data that analyzed both germline arms used the average number of cell corpses per animal. This step was not included for experiments where only one germline arm was analyzed. Normality of the data was assessed using a Shapiro–Wilk test. Since all datasets were found to include non-normal data, a nonparametric pairwise Wilcoxon *t*-tests with the Benjamani and Hochberg correction was performed to determine which data were significantly different from each other. All analysis was performed in RStudio using R—4.3.3 (R Core Team 2023; Posit Team 2025). For all analyses, a *P*-value cutoff of ≤ 0.05 was considered significant with an indication on graphs if the *P*-value was also ≤ 0.01.

### Oocyte size and number analysis

Worms were grown to the L4 stage at 20 °C, then isolated and maintained at 20 °C or upshifted to 26 °C for 24 h prior to imaging. For imaging, worms were mounted on slides with 2% agarose pads in 1 mM levamisole in 1X M9, without bacteria. Oocytes from 1 gonad arm were imaged from each worm using a Nikon Eclipse TE2000-S inverted microscope equipped with a Plan Apo 60x/1.25 numerical aperture oil objective. Images were captured using a Q imaging Exi Blue camera (Teledyne Photometrics, Tucson, AZ, USA) using Nomarski optics and Q Capture Pro 7 software (Teledyne Photometrics, Tucson, AZ, USA). The area of each fully cellularized oocyte within 1 germline arm was measured in FIJI using the freehand tool with a scale of 4.64 in by 3.47 in (Schindelin et al. 2012). The area was measured by tracing around the membrane of each of the fully cellularized oocytes. Oocytes were considered fully cellularized if they had a cell membrane fully surrounding the nucleus. Three to 5 biological replicates were performed, with 3 to 6 worms analyzed per replicate for a total of *n* = 17 to 27 germline arms per genotype per temperature. Statistical analysis was done using a 2-way ANOVA with Tukey's multiple comparisons using Prism 10.0.3 (GraphPad, Boston, MA, USA).

### Ovulation rate analysis

Ovulation rate analysis was performed as in Rios et al. (2017). Worms were grown to the L4 stage at 20 °C, then isolated and maintained at 20 °C or upshifted to 26 °C for 24 h prior to analysis. On the day of the ovulation assay, adult worms were cloned to a plate with a thin lawn of *E. coli* and the embryos inside the uterus were immediately counted. Worms were then allowed to lay embryos for 3 h at the appropriate temperature. At the end of the 3 h, the embryos inside the uterus were counted, then the worm was removed from the plate, and the embryos laid on the plate were counted. All steps were visualized on a Nikon SMZ1500 stereomicroscope. We used the following formula to calculate the ovulation rate per gonad arm per hour: ([Final # embryos in the uterus—Initial # embryos in the uterus] + Number of embryos on the plate)/(2 gonads × 3 h). Seven to 10 biological replicates were performed, with 1 to 3 worms analyzed per replicate for a total of *n* = 16 to 26 worms per genotype. Statistical analysis was done using a 2-way ANOVA using with Tukey's correction using Prism 10.0.3 (GraphPad, Boston, MA, USA).

### Cytoplasmic streaming analysis

Cytoplasmic streaming analysis was performed as in Wolke et al. (2007). Worms were grown to the L4 stage at 20 °C, then isolated and maintained at 20 °C or upshifted to 26 °C for 24 h prior to imaging. Immediately before imaging, all worms were anesthetized in a 1 mM levamisole in 1X M9 buffer solution for 5 min in a glass well and then placed on a 4% agarose pad while maintained in the levamisole. Worms were imaged every 15 s over 20 min using Nomarski optics on a Nikon Eclipse TE2000-S inverted microscope equipped with a Plan Apo 60X/1.25 numerical aperture oil objective. Images were captured using a Q imaging Exi Blue camera (Teledyne Photometrics, Tucson, AZ, USA) and Q Capture Pro 7 software (Teledyne Photometrics, Tucson, AZ, USA). To measure the speed of cytoplasmic streaming, particles within the rachis were chosen that were approaching or traveling around the germline bend and were visible within the focal plane for a minimum of 2 min. Each particle was tracked over 2 min using the “Manual Tracking” plugin in Fiji (Schindelin et al. 2012). For each genotype and temperature, 5 particles in each of 6 independent germlines were analyzed. Statistical analysis was done using 2-way ANOVA with Tukey's correction using Prism 10.0.3 (GraphPad, Boston, MA, USA).

### Germline length analysis

Worms were grown to the L4 stage at 20 °C, then isolated and maintained at 20 °C or upshifted to 26 °C for 24 h prior to imaging. For imaging, worms were mounted on slides with 2% agarose pads in 1 mM levamisole in 1XM9, without bacteria. The whole worm body was imaged using Leica Application Suite Advanced Fluorescence 3.2 software using Leica CTR6000 deconvolution inverted microscope with a Hamatsu Orca-R2 camera, and the 10X HCX PL Fluotar 0.3 NA objective lens and one germline arm was imaged using a 40X HCX PL Fluotar 0.75 NA objective using Nomarski optics. Whole-body length and germline length were measured in FIJI using the free hand line tool (Schindelin et al. 2012). When more than 1 image was required, the pairwise stitching plugin in FIJI was used to stitch images (Preibisch et al. 2009). Whole worm lengths were measured from the nose to anus. Germline length was measured from distal tip to the spermatheca. The normalized germline length was calculated by the following equation: (germline length [mm])/(whole worm length [mm]) = normalized germline length. Two to 4 biological replicates were performed, with 3 to 14 worms analyzed per replicate for a total of *n* = 20 to 24 worms per genotype per temperature. Statistical analysis was done using a 2-way ANOVA with Tukey's multiple comparison using Prism 10.0.3 (GraphPad, Boston, MA, USA).

## Results

### LIN-35 and DREAM complex mutants do not fully induce germline apoptosis in response to moderate temperature stress

Given the known roles of LIN-35 in promoting germline apoptosis under other conditions (Schertel and Conradt 2007; Láscarez-Lagunas et al. 2014), we investigated whether LIN-35 promotes increased germline apoptosis levels during moderate temperature stress at 26 °C. As LIN-35 is known to interact with the DREAM complex and has been shown to co-bind with the DREAM complex at the *ced-9* operon (Kudron et al. 2013; Goetsch et al. 2017), we also investigated whether members of the Muv B core of the DREAM complex, LIN-54 and LIN-37, could also promote germline apoptosis (Fig. 1a). We chose to investigate LIN-54 because it is the DNA-binding protein of the Muv B core, and the *lin-54(n2231)* hypomorph has been shown to have highly disrupted DREAM complex chromatin binding while still being fertile (Harrison et al. 2006; Tabuchi et al. 2011). We chose to investigate LIN-37 because, along with LIN-35, it has previously been shown to have pro-apoptotic function in the soma (Reddien et al. 2007) and the *lin-37(n758)* hypomorph is fully fertile. To determine the level of apoptosis, we used strains that carried the CED-1::GFP transgene, which allows for visualization of germline apoptotic cells (Zhou et al. 2001; Schumacher et al. 2005). We counted apoptotic cells in wild type and the 3 mutant strains, *lin-35(n745)*, *lin-54(n2231)*, and *lin-37(n758)*, under 3 temperature conditions: continual exposure to 20 °C (nonstress condition), upshifting to 26 °C at the L1 stage (temperature stress across all germline development), and up-shifting to 26 °C at the L4 stage (temperature stress after most germline development). We assessed the number of apoptotic cells in worms 24 h post-L4 stage. Wild-type, but not mutant, hermaphrodites showed a significant increase in apoptosis when worms were upshifted at the L1 stage compared with the same genotype maintained at 20 °C (Fig. 1b). On the other hand, all genotypes showed a significant increase in apoptosis when hermaphrodites were upshifted at the L4 stage compared with the same genotype maintained at 20 °C (Fig. 1b). However, all 3 mutants showed a significantly lower level of apoptosis than wild type for both 26 °C stress conditions (Fig. 1b). These data suggest a role for LIN-35 and the DREAM complex Muv B core in the induction of germline apoptosis in response to moderate temperature stress.

### Constitutively active CED-9 mutants do not induce apoptosis to wild-type levels in response to moderate temperature stress

Previous research has shown that expression of *ced-9* is regulated by LIN-35 during DNA damage and starvation-induced apoptosis (Schertel and Conradt 2007; Láscarez-Lagunas et al. 2014). This suggests that *ced-9* mRNA levels, and likely CED-9 protein levels, could play a role in induction of germline apoptosis during multiple stress conditions. To further investigate the potential role of CED-9 regulation in stress-induced germline apoptosis, we used the *ced-9(n1950)* mutation, which constitutively sequesters CED-4 leading to a block in activation of apoptosis (Fig. 1a) (Hengartner and Horvitz 1994). Above, we found that upshifting hermaphrodites to 26 °C at the L4 stage had the strongest effect on the activation of germline apoptosis (Fig. 1b); therefore, we used this temperature treatment for all of our subsequent experiments. We found that *ced-9(n1950)* mutants showed a smaller increase in apoptosis compared to wild-type animals, similar to levels seen in *lin-35* and DREAM complex mutants with the same temperature treatment (Fig. 1c). We next measured the level of apoptosis in *ced-9(n1950); lin-54(n2231)* double mutants using the same temperature treatment. We found that the double mutant had no induction of apoptosis during moderate temperature stress (Fig. 1c). Consistent with previously published data (Gumienny et al. 1999), the level of apoptosis did not go to zero in either mutant containing *ced-9(n1950)* under any conditions. Thus, like DNA damage and asynapsis-induced apoptosis (Gartner et al. 2000; Ye et al. 2014), repression of CED-9 function is important for the increase in apoptosis during moderate temperature stress, but not for physiological apoptosis.

### Activation of the synapsis checkpoint, but not the DNA damage checkpoint, is necessary for an increase in apoptosis during temperature stress

There are 2 well-established checkpoints that, when triggered, lead to an increase in apoptosis in the germline: the DNA damage checkpoint mediated by CEP-1/p53 and the synapsis checkpoint mediated by BUB-3 and PCH-2 (Fig. 1a) (Schumacher et al. 2001; Bhalla and Dernburg 2005; Bohr et al. 2015). To determine if either checkpoint is required for the increase in germline apoptosis, we looked at the level of apoptosis in mutants in *cep-1(gk138), bub-3(ok3437),* and *pch-2(tm1458).* If one of the checkpoints were necessary, we would predict to see no increase in apoptosis in the corresponding mutant at 26 °C. In *cep-1(gk138)* mutants, which lack a DNA damage checkpoint, we saw an increase in apoptosis indistinguishable from wild type (Fig. 1d). On the other hand, in *bub-3(ok3437)* and *pch-2(tm1458)* mutants, which lack a synapsis checkpoint, we saw either no or a minimal increase in apoptosis at 26 °C respectively (Fig. 1d). Additionally, the level of apoptosis in *bub-3(ok3437)* and *pch-2(tm1458)* mutants at 26 °C was indistinguishable from wild type at 20 °C (Fig. 1d). This work demonstrates that the synapsis checkpoint, but not the DNA damage checkpoint, is necessary for increased apoptosis during moderate temperature stress.

### LIN-35 and DREAM complex mutants do not show increased cytoplasmic streaming in response to moderate temperature stress

**Fig. 2.**
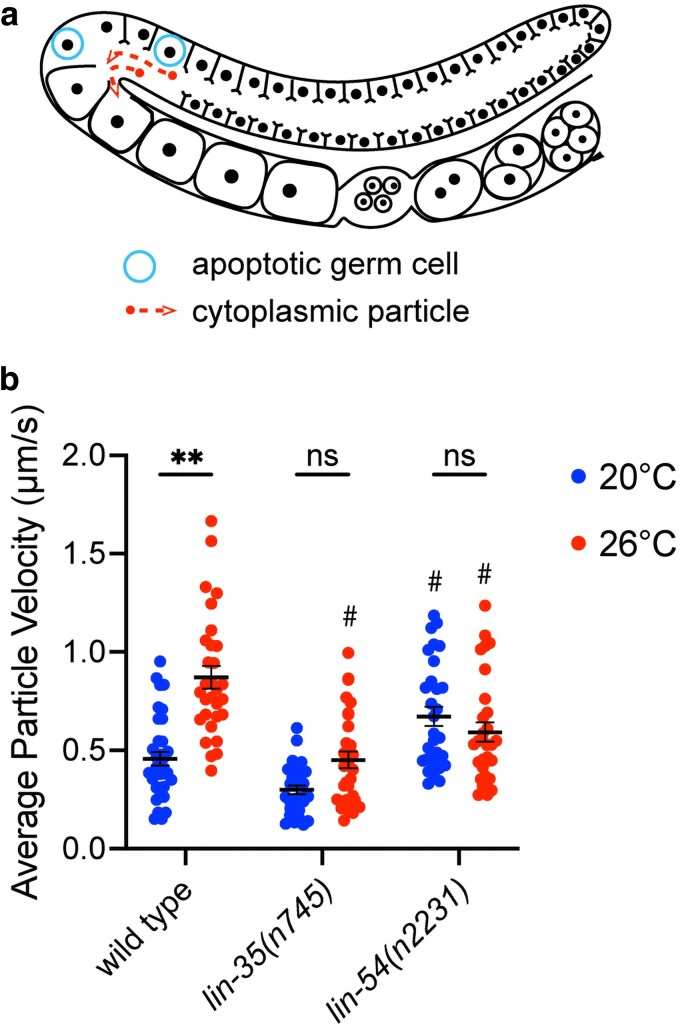
LIN-35 and DREAM complex members are necessary for increased cytoplasm streaming at during temperature stress. a) Model of 1 arm of an oogenic germline. Apoptotic germ cells being engulfed are represented by blue circles. Cytoplasmic streaming is represented by the trajectory of the red dots. b) The rate of cytoplasmic particle movement was measured under DIC microscopy in wild type, *lin-35(n745)*, and *lin-54(n2231)* mutants. Hermaphrodites were either maintained continually at 20 °C (blue) or upshifted to 26 °C at the L4 stage (red) with each dot representing an individual particle tracked, *n* = 5 particles per 6 gonads for a total of 30 particles per genotype tracked. **P ≤ 0.01 significantly different within the genotype compared to 20 °C, #*P* ≤ 0.05 significantly different than wild type at the same temperature using 2-way ANOVA with Tukey’s correction. Error bars indicate ±SEM.

One of the functions of germline apoptosis is the contribution of cytoplasmic components from the dying nuclei to developing oocytes (Gartner et al. 2008). In *C. elegans,* cytoplasm that is expelled from dying nuclei into the central core of shared cytoplasm (rachis) moves around the bend of the germline into cellularizing oocytes in a process called cytoplasmic streaming (Fig. 2a; Wolke et al. 2007). In addition, while apoptosis is not required for cytoplasmic streaming to occur, the rate of cytoplasmic streaming has been shown to depend on apoptosis (Wolke et al. 2007). Since temperature generally increases the rate of many cellular and physiological processes, we investigated changes in cytoplasmic streaming in wild type, *lin-35(n745),* and *lin-54(n2231)* mutants at different temperatures. We found that the rate of cytoplasmic streaming was significantly higher in wild-type worms upshifted to 26 °C compared to wild-type worms maintained at 20 °C (Fig. 2b). In contrast, we found no change in the rate of cytoplasmic streaming in *lin-35(n745)* or *lin-54(n2231)* mutants upshifted to 26 °C compared to the same genotype maintained at 20 °C (Fig. 2b). This failure to increase cytoplasmic streaming in the mutants when upshifted to 26 °C corresponded with their failure to increase apoptosis to the same level as wild type under the same temperature conditions. We also found that *lin-54(n2231)* mutants had a significantly higher rate of cytoplasmic streaming at 20 °C than that of wild type at 20 °C (Fig. 2b). On the other hand, both *lin-35(n745)* and *lin-54(n2231)* mutants had significantly slower rates of cytoplasmic streaming when upshifted to 26 °C than that of wild type upshifted to 26 °C (Fig. 2b). These data are consistent with a model where increased apoptosis under temperature stress contributes to increased cytoplasmic streaming. Increased cytoplasmic streaming during temperature stress could, in turn, lead to oocytes similar in sized to those in unstressed animals accompanied by a faster ovulation rate or the production of larger oocytes than in unstressed animals.

### Temperature does not affect ovulation rate in wild-type or mutants

**Fig. 3.**
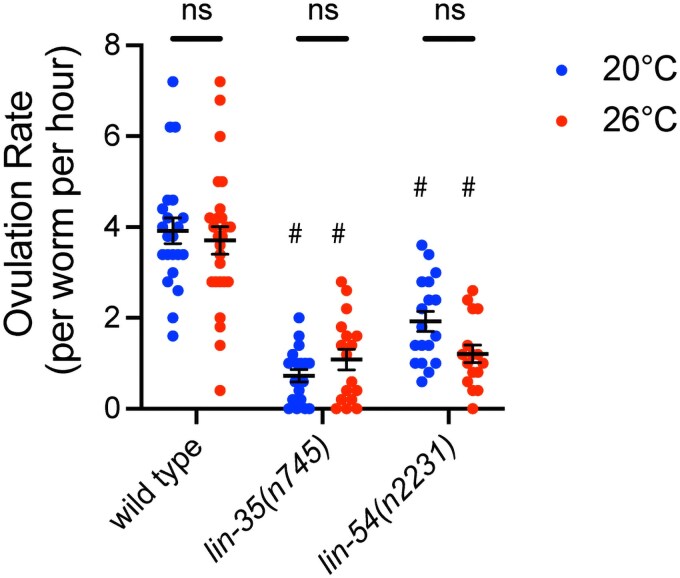
Ovulation rate does not change in wild type, *lin-35*, or DREAM complex mutants during temperature stress. Ovulation rates measured in wild type, *lin-35(n745),* and *lin-54(n2231)* mutants. Hermaphrodites were either maintained continually at 20 °C (blue) or upshifted to 26 °C at the L4 stage (red) with each dot representing the ovulation rate within an individual worm, *n* = 16 to 26 worms per genotype. ns, not significantly different within the genotype compared with 20 °C, #*P* ≤ 0.05 significantly different than wild type at the same temperature using 2-way ANOVA with Tukey’s correction. Error bars indicate ±SEM.

We investigated if ovulation rate increased when worms were exposed to moderate temperature stress. We found that neither wild type nor mutant adult worms demonstrated an increase in ovulation rate when upshifted to 26 °C at the L4 stage compared with the same strain at 20 °C (Fig. 3). However, both *lin-35(n745)* and *lin-54(n2231)* mutants had significantly slower ovulation rates than wild type at the same temperature (Fig. 3). These data suggest that neither temperature nor apoptosis level directly contributed to the rate of ovulation. However, the pleotropic defects in the germline experienced in *lin-35(n745)* and *lin-54(n2231)* mutants (Goetsch et al. 2017; Mikeworth et al. 2023) seem to affect overall ovulation rate.

### Oocyte size increases with moderate temperature stress

**Fig. 4.**
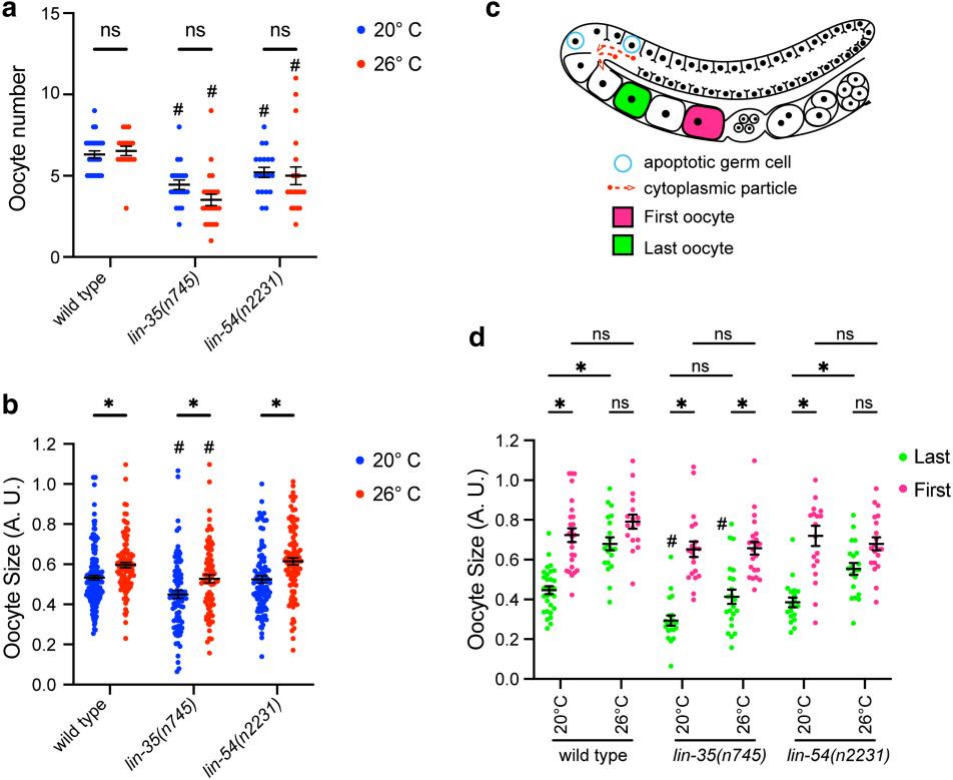
Oocyte size but not number changes in wild type, *lin-35*, and DREAM complex mutant during temperature stress. a) Oocyte number was scored in wild type, *lin-35(n745)*, and *lin-54(n2231)* mutants. Hermaphrodites were either maintained continually at 20 °C (blue) or upshifted to 26 °C at the L4 stage (red) with each dot representing the oocyte number within a gonad arm, *n* = 15 to 26 gonad arms per genotype per temperature. b) Individual oocyte sizes were measured in wild type, *lin-35(n745)*, and *lin-54(n2231)* mutants. Hermaphrodites were either maintained continually at 20 °C (blue) or upshifted to 26 °C at the L4 stage (red) with each dot representing an individual oocyte size, *n* = 81 to 165, across 15 to 26 worms per genotype per temperature. c) Model of the germline with the last (green) and first (pink) oocytes labeled. d) The measurements of the last (green) and first (pink) oocyte sizes were pulled out of the data from (b) for wild type, *lin-35(n745)*, and *lin-54(n2231)* mutants at 20 °C or upshifted to 26 °C at the L4 stage (red) with each dot representing an individual oocyte size, *n* = 17 to 30, across 15 to 26 worms per genotype per temperature. *P ≤ 0.05, **P ≤ 0.01 significantly different within the genotype compared with 20 °C or (d only) between the same oocyte within genotype and temperature, #*P* ≤ 0.05 significantly different than wild type at the same temperature using 2-way ANOVA with Tukey correction. Error bars indicate ±SEM.

We next investigated if the number or size of oocytes was affected when worms were exposed to postdevelopmental temperature stress. We found that neither wild type nor mutant adult worms demonstrated a change in the number of oocytes present when upshifted to 26 °C at the L4 stage compared with the same strain at 20 °C (Fig. 4a). However, both *lin-35(n745)* and *lin-54(n2231)* mutants did show a nonsignificant trend toward fewer oocytes when upshifted to 26 °C compared with the same strain at 20 °C (Fig. 4a). In addition, both *lin-35(n745)* and *lin-54(n2231)* mutants had significantly lower numbers of oocytes than wild type at the same temperature, with the exception of *lin-54(n2231)* at 20 °C (Fig. 4a). We also found that both wild type and mutant worms had, on average, larger oocytes when upshifted to 26 °C compared with the same strain at 20 °C (Fig. 4b). However, *lin-35(n745)* mutants had significantly smaller oocytes than wild type at the same temperature (Fig. 4b). To determine if the changes we see in mutants in the level of germline apoptosis or oocyte number are due to general changes in germline length, we measured germline length in *lin-35(n745), lin-54(n2231),* and *ced-9(n1950)* mutants. We found that normalized germline lengths did not differ in either wild type or mutants during temperature stress at 26 °C compared with 20 °C (Supplementary Fig. 1). We also did not see any difference in normalized germline length between wild type and any of the mutants (Supplementary Fig. 1).

We next wanted to know if the change in the overall size of oocytes was driven primarily by increased cytoplasmic stream during moderate temperature stress. Oocyte growth occurs through 2 mechanisms. First, oocytes gain a large proportion of their size during cellularization through cytoplasmic streaming (Wolke et al. 2007). Second, after cellularization has occurred, additional oocyte growth occurs through the import of yolk lipoproteins that are produced in the intestine (Greenstein 2005). Thus, we compared the last oocyte in the line (the one most recently cellularized) and the first oocyte in the line (the one next to be ovulated) from the dataset used in Fig. 4b (Fig. 4c). We found that in wild-type worms, there was a significant size difference between the first and last oocyte at 20 °C, while at 26 °C, there was no significant difference in size between the first and last oocyte. There was a similar pattern in the sizes of the first and last oocytes in the *lin-54(n2231)* mutants as seen in wild type. However, in *lin-35(n745)* mutants, there was a similar difference in size between the first and last oocyte at both temperatures (Fig. 4d). The last oocyte, but not the first oocyte, in *lin-35(n745)* mutants alone was significantly smaller than the same oocyte class in wild type at both temperatures (Fig. 4d). These data suggest that the overall increased size in oocytes seen during moderate temperature stress is primarily driven by oocytes being bigger at the point that they are first cellularized.

## Discussion

Germ cells that make oocytes have the combined need to provide to the 1-cell embryo an intact genome and sufficient, high-quality cytoplasmic components. We have shown that increasing germline apoptosis during moderate temperature stress can aid in both processes. Here, we found that components of the DREAM complex, LIN-35 and the Muv B core proteins LIN-54 and LIN-37, are necessary for the full increase in apoptosis seen during moderate temperature stress. Similarly, repression of CED-9 protein function is necessary for the full increase in apoptosis seen during moderate temperature stress. However, induction of germline apoptosis during moderate temperature stress is completely abolished when LIN-54 DNA-binding activity is reduced and CED-9 is constitutively active, suggesting these 2 pathways work together to regulate apoptosis during moderate temperature stress. In addition, increased apoptosis during moderate temperature stress is also completely reliant on the synapsis checkpoint, but not the DNA damage checkpoint. Finally, we found that the rate of cytoplasmic streaming and the size of oocytes increase in wild type during moderate temperature stress. These findings expand the known role of LIN-35 and CED-9 and add the Muv B core as central regulators of stress-induced germline apoptosis.

### The DREAM complex with LIN-35 are core regulators of apoptosis through regulation of *ced-9* expression

An accumulation of data suggests a broad role for the DREAM complex, including the Muv B core and LIN-35, in the regulation of the level of germline apoptosis. LIN-35 has been shown to be necessary to maintain the moderate level of physiological germline apoptosis and functions in activation of apoptosis in starvation stress and DNA damage (Schertel and Conradt 2007; Láscarez-Lagunas et al. 2014). We have added moderate temperature stress to this list of apoptosis regulation by LIN-35. We also found that proteins in the Muv B core are equally necessary for activation of apoptosis during moderate temperature stress. Previous work has shown that the expression level of *ced-9* is repressed by LIN-35 during both physiological apoptosis and starvation stress in the *C. elegans* germline (Schertel and Conradt 2007; Láscarez-Lagunas et al. 2014). Given that LIN-35 cannot bind DNA and regulate target genes without a DNA-binding partner, it seems likely that it is through its interaction with the Muv B core of the DREAM complex that LIN-35 functions to regulate *ced-9* expression. This is supported by the fact that the components of the Muv B core and LIN-35 have been shown to bind the *ced-9* operon (Goetsch et al. 2017). In addition, the other major LIN-35-binding partner that functions in DNA binding, E2F, has been shown to function distinctly from LIN-35 in the germ line, including in regulation of apoptosis (Chi and Reinke 2006; Schertel and Conradt 2007; Kudron et al. 2013; Láscarez-Lagunas et al. 2014). This function of Rb/pocket protein homologs repressing expression of anti-apoptotic BCL2 proteins seems to be conserved in at least *Drosophila melanogaster.* The Rb/LIN-35 homolog Rbf1 has been shown to repress the expression of the anti-apoptotic BCL2/CED-9 homolog *buffy* to promote apoptosis in the *Drosophila* wing disc in a manner similar to what is seen in the *C. elegans* germline (Clavier et al. 2014). While DREAM has been extensively studied in mammals, a clear role in apoptosis regulation outside of its regulation of the cell cycle has not been clearly demonstrated yet (Hoareau et al. 2024). It has not yet been tested if other forms of cellular stress that also induce apoptosis, such as oxidative stress, osmotic stress, and heat shock, are also promoted through the LIN-35/DREAM complex. However, it would be informative to test these pathways to determine how universal the role of the LIN-35/DREAM complex is in germline apoptosis. Overall, there seems to be a strong case, in at least invertebrates, for Rb/LIN-35 and DREAM to be a core regulator of apoptosis levels through regulation of *ced-9* transcription.

### Parallel regulation of apoptosis distinguishes how apoptosis is triggered under different stressors

**Fig. 5.**
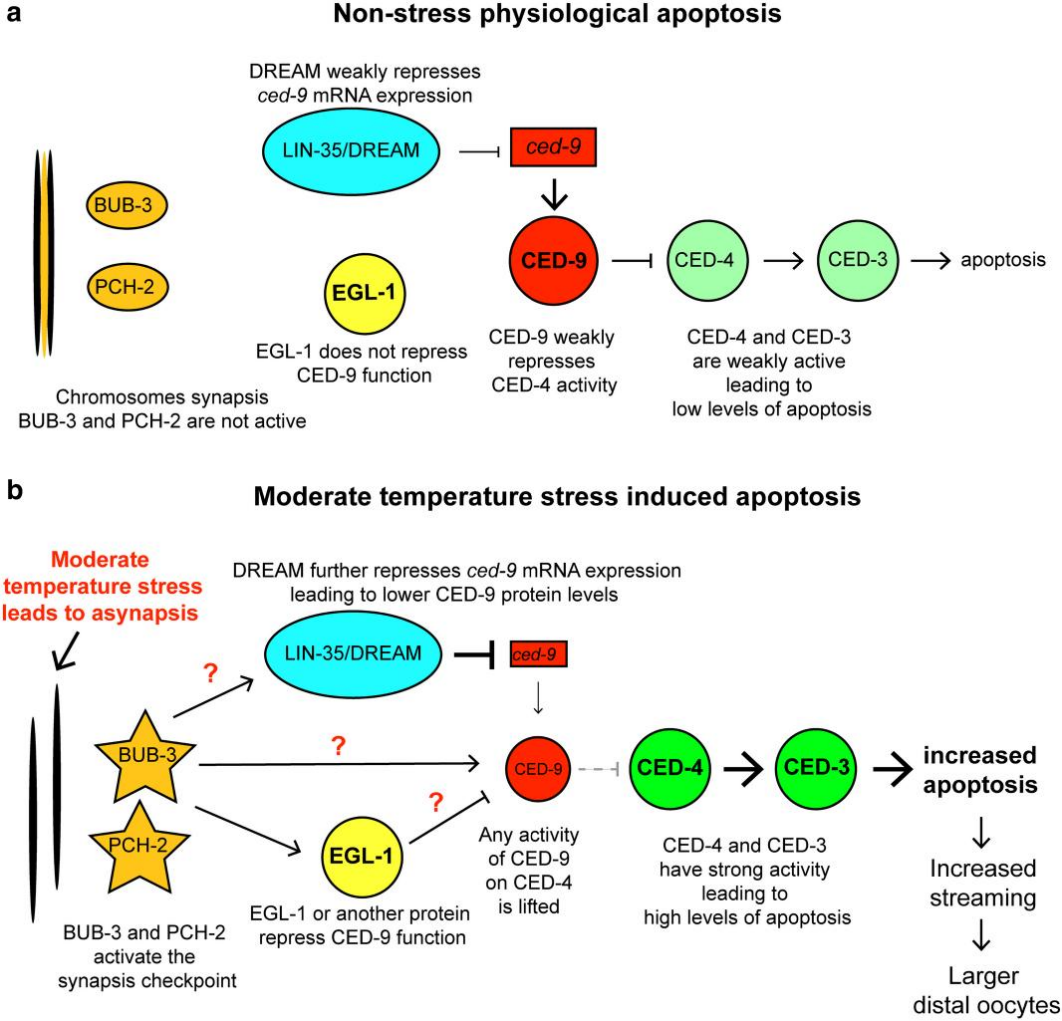
Model of apoptosis induction during moderate temperature stress. a) Under nonstress conditions, apoptosis levels are at physiological conditions. Chromosomes synapse properly and the BUB-3/PCH-2 synapsis checkpoint is not activated. LIN-35 and the DREAM complex weakly repress *ced-9* expression (Schertel and Conradt 2007), leading to moderate levels of physiological apoptosis. Physiological apoptosis is not dependent on EGL-1 (Gumienny et al. 1999). b) Moderate temperature stress leads to increased asynapsis, which leads to activation of the synapsis checkpoint through BUB-3 and PCH-2 (Bhalla and Dernburg 2005; Bilgir et al. 2013; this study). LIN-35 and the DREAM complex likely further repress the expression of *ced-9* leading to decreased levels of CED-9 protein. Through EGL-1 or some other activity, CED-9 binding of CED-4 is also relieved, leading to high CED-4 activation of the CED-3 caspase and increased levels of apoptosis. Higher rates of apoptosis lead to increased cytoplasmic streaming and larger oocytes at the point of cellularization.

While it seems likely that the LIN-35/DREAM complex functions to promote apoptosis through regulation of *ced-9* expression during different stressors, there are varied pathways that likely work in parallel to promote activation of apoptosis under these different stressors. For example, LIN-35 is necessary for the increase in germline apoptosis during DNA damage, starvation stress, and moderate temperature stress (Schertel and Conradt 2007; Láscarez-Lagunas et al. 2014, this study). But across these 3 stressors, there are different effects of other known mediators of apoptosis: (i) the increase in apoptosis during DNA damage is lost in both p53/*cep-1* loss-of-function (lf) mutants and BCL2/*ced-9* gain-of-function (gf) mutants (Gartner et al. 2000; Schertel and Conradt 2007), (ii) there is no effect on increased apoptosis in starvation stress with either p53/*cep-1(lf)* mutations or BCL2/*ced-9(gf)* mutations (Salinas et al. 2006), and (iii) we see loss of increased apoptosis during moderate temperature stress with BCL2/*ced-9(gf)* mutants but not p53/*cep-1(lf)* mutants (this study). These different effects of *cep-1(lf)* and *ced-9(gf)* mutants point toward different pathways that likely regulate apoptosis during these different stressors in parallel to LIN-35/DREAM activity. In all 3 stressors, LIN-35/DREAM likely functions to downregulate the level of *ced-9* expression, leading to cells primed for increased apoptosis. In the case of DNA damage, p53/CEP-1 activation of EGL-1 levels leads to a further decrease of CED-9 repression of CED-4 function (Gartner et al. 2000; Schertel and Conradt 2007). In the case of starvation stress, in addition to downregulation of *ced-9* RNA and protein, there is concurrent upregulation of *ced-4* expression mediated through DPL-1 (Láscarez-Lagunas et al. 2014). In the case of moderate temperature stress, we have shown that upregulation of apoptosis is dependent on the synapsis checkpoint (Fig. 1d, Fig. 5). Like what has been previously shown for synapsis checkpoint mutants, this increase in apoptosis is not dependent on p53/CEP-1 (Fig. 1d; Bhalla and Dernburg 2005). There are no current data investigating if apoptosis activated through the synapsis checkpoint is decreased in *ced-9(gf)* mutants. However, the synapsis checkpoint does function through a p53/CEP-1 independent activation of *egl-1* expression, which would likely work through inactivation of CED-9 protein function (Ye et al. 2014). Thus, we predict that the decrease in moderate temperature stress-induced apoptosis we see in *ced-9(gf)* mutants is through a synapsis checkpoint-mediated increase in *egl-1* expression (Fig. 5). Additionally, a fourth pathway, MAPK signaling, has been shown to be important for upregulation of apoptosis for other stressors such as oxidative stress, osmotic stress, and heat shock (Salinas et al. 2006). Thus, cells use a variety of stress-type specific mechanisms to promote apoptosis in addition to regulation of *ced-9* expression.

An important aspect of germline stress-induced apoptosis that is unexplored is what mechanisms lead to a change in LIN-35/DREAM complex regulation of *ced-9* expression. These proteins are already both expressed in the germline and regulate *ced-9* expression levels under nonstress conditions, as has been shown by the loss of *lin-35* increasing physiological apoptosis levels (Schertel and Conradt 2007). In the case of starvation stress, expression of *lin-35* is known to increase (Láscarez-Lagunas et al. 2014), which may result in a stronger repression of *ced-9* expression. However, the function of the DREAM complex in other contexts has been shown to be highly regulated through post-transcriptional modifications; in particular, phosphorylation of various DREAM complex components (Engeland 2022). This leaves open other cellular methods to regulate the repressive function of the LIN-35/DREAM complex. Further studies to determine if there are conserved or differing mechanisms that differentially regulate the strength of the LIN-35/DREAM complex *ced-9* repression during different stresses are an interesting area of investigation that could lead to a more complete picture of germline apoptosis regulation.

### Apoptotic induction due to elevated temperatures differs based on the type of heat stress applied

In our work, we have focused on temperature stress treatments that are long in duration (24 + hours) and at a moderate temperature of 26 °C, which lies just below the temperature threshold that results in species sterility (27 °C) (Harvey and Viney 2007; Petrella 2014; Poullet et al. 2015). These temperature treatments have effects on fertility and induce apoptosis, but are well below the temperatures studied to induce canonical heat shock response (≥33 to 37 °C) (Zevian and Yanowitz 2014). Previous work has shown that temperature treatments of short duration (1 hr) and more extreme temperatures (33 °C) strongly induce the heat shock response and also induce germline apoptosis (Hajdu-Cronin et al. 2004; Salinas et al. 2006). Unlike what we show here for moderate temperature stress, apoptosis induced by heat shock is not decreased in a *ced-9(gf)* mutants (Salinas et al. 2006). This suggests that heat shock-induced germline apoptosis is not due to activation of the synapsis checkpoint but rather signaling that is part of the canonical heat shock response. Moderate temperature stress leading to activation of the synapsis checkpoint is consistent with previous work that has shown that temperatures ≥26.5 °C leads to failure of synaptonemal complex assembly and increased asynapsis (Bilgir et al. 2013; Rog et al. 2017). In our experiments, we also saw there was a higher level of apoptosis induced in animals exposed to moderate temperature stress starting at the L4 stage when compared with animals where the moderate temperature stress started at the L1 stage. This is consistent with data that assembly of the synaptonemal complex may be able to adapt over time to moderate temperature stress (Bilgir et al. 2013). Thus, there may be lower levels of unsynapsed chromosomes in germlines that have completely developed at 26 °C. In general, temperatures at or above the thermal threshold of fertility lead to cytotoxicity-independent DNA damage. However, depending on the severity and type of the thermal stress, germ cells use different mechanisms to mediate removal of damaged cells through apoptosis.

Finally, throughout the experiments where we have looked at levels of apoptosis, we have used the *ced-1::gfp* transgene to measure apoptosis. The use of the *ced-1::gfp* transgene is commonplace for the evaluation of germline apoptosis (Salinas et al. 2006; Morrison et al. 2014; Fausett et al. 2021); however, it is possible that this transgene reports delayed engulfment of apoptotic nuclei, and not an increase in apoptosis levels. To fully rule out the possibility that there are moderate temperature stress solely or primarily changes the rate of apoptotic cell engulfment, further experiments would need to be done using other methods to visualize apoptosis. However, we feel our data showing the effects of known synapsis checkpoint mutants results in limited to no increase in apoptosis 26 °C compared with 20 °C suggest that there is a real increase in apoptosis during moderate temperature stress due to increased asynapsis.

### A role for apoptosis in preserving oocyte quality during stress through changes in cytoplasmic streaming

Previous research has shown that a decrease in apoptosis led to decreased cytoplasmic streaming and embryonic size (Wolke et al. 2007; Fausett et al. 2021). We saw the concomitant increase in streaming during moderate temperature stress when apoptosis is increased in wild type. The increase in streaming seen in wild type correlated with an increase in oocyte size in the last cellularized oocyte during moderate temperature stress, suggesting that the increase in streaming leads to a larger volume of cytoplasm in oocytes. However, the first oocyte, the oocyte that is next to be ovulated, was not bigger during moderate temperature stress compared with nonstress conditions. Since oocyte growth after cellularization is primarily driven by import of yolk lipoprotein (Greenstein 2005), the lack of growth in the first oocyte could represent either a lower transport of yolk during moderate temperature stress or an overall constraint on oocyte growth due to the somatic gonad precluding oocytes from expanding past a specific size. In either case, oocytes are likely getting a larger percentage of their cytoplasmic volume from germline cytoplasmic streaming during moderate temperature stress than during nonstress conditions. The higher percentage of germline-sourced cytoplasm in oocytes could include larger amounts of organelles, ribosomes, and germline-expressed mRNAs and proteins that could positively or negatively affect embryos formed during moderate temperature stress. Work from other labs has showed that oocyte quality during/after stress is better if germline apoptosis is present (Andux and Ellis 2008; Fausett et al. 2021). In both aging and after acid, oxidative, starvation, and ethanol stress, there was a significant increase in embryonic lethality in *ced-3* and *ced-4* mutants that eliminate all germline apoptosis (Andux and Ellis 2008; Fausett et al. 2021). In both these studies, the authors also saw a decrease in oocyte size in *ced-3* mutants during aging and after stress, respectively (Andux and Ellis 2008; Fausett et al. 2021), similar to what we see in *lin-35* mutants after moderate temperature stress. This suggests that increased oocyte provisioning through apoptosis-driven cytoplasmic streaming could play a role in preserving embryo quality during/after a significant germline stress.

Finally, it is interesting to note that our findings on the level of induction of apoptosis are discordant with data on moderate temperature stress and brood size. In previous studies, chronic exposure to moderate temperature stress starting at the embryo or L1 stage has a significantly stronger effect on brood size compared with a shorter exposure starting at the L4 stage (Cherian et al. 2020; Mikeworth et al. 2023), which is the opposite of what we see with apoptosis levels. The most likely explanation of decreased brood size with longer heat exposure is that it is the result of additional effects of moderate temperature stress on germline development that limit fertility, but do not directly lead to induction of apoptosis. However, a reduced level of apoptosis at temperatures that are still stressful could also lead to poorer oocyte quality and contribute to the smaller brood sizes.

## Supplementary Material

jkaf228_Supplementary_Data

## Data Availability

Strains are available upon request. All raw data and images are available at GSA online and https://epublications.marquette.edu/compere2025/. The raw data in Supplementary Tables 1 to 4 include all raw apoptosis counts, all raw streaming velocities, raw ovulation counts, raw oocyte measurements and counts, and all raw *P*-values. Each type of data is available as a spreadsheet associated with a particular figure. Supplemental material available at G3 online.
