# Supplementary material for: The synapsis checkpoint and the LIN-35/DREAM complex promote temperature stress-induced increases in germline apoptosis in Caenorhabditis elegans
Source: G3 (Bethesda). 2025 Sep 26;15(12):jkaf228. doi: 10.1093/g3journal/jkaf228 (PMC12693621; doi:10.1093/g3journal/jkaf228)
Supplement: jkaf228_Supplementary_Data [file jkaf228_supplementary_data.zip › Supplemental_Figure_1_Legend_G3-2025-406020.docx]

**Supplemental Figure 1: Germline length is consistent across mutants and temperatures**

Germline length normalized to worm length in wild type, *lin-35(n745),* *lin-54(n2231)* and *ced-9(n1950)* mutants. Hermaphrodites were either maintained continually at 20°C (blue) or upshifted to 26°C at the L4 stage (red) with each dot representing the normalized length of a gonad arm, n = 20-24 worms per genotype per temperature. ns = not significantly different within the genotype compared to 20°C using two-way ANOVA with Tukey correction. Error bars indicate + SEM.
