# Supplementary figures and images for: The synapsis checkpoint and the LIN-35/DREAM complex promote temperature stress-induced increases in germline apoptosis in Caenorhabditis elegans
Source: G3 (Bethesda). 2025 Sep 26;15(12):jkaf228. doi: 10.1093/g3journal/jkaf228 (PMC12693621; doi:10.1093/g3journal/jkaf228)

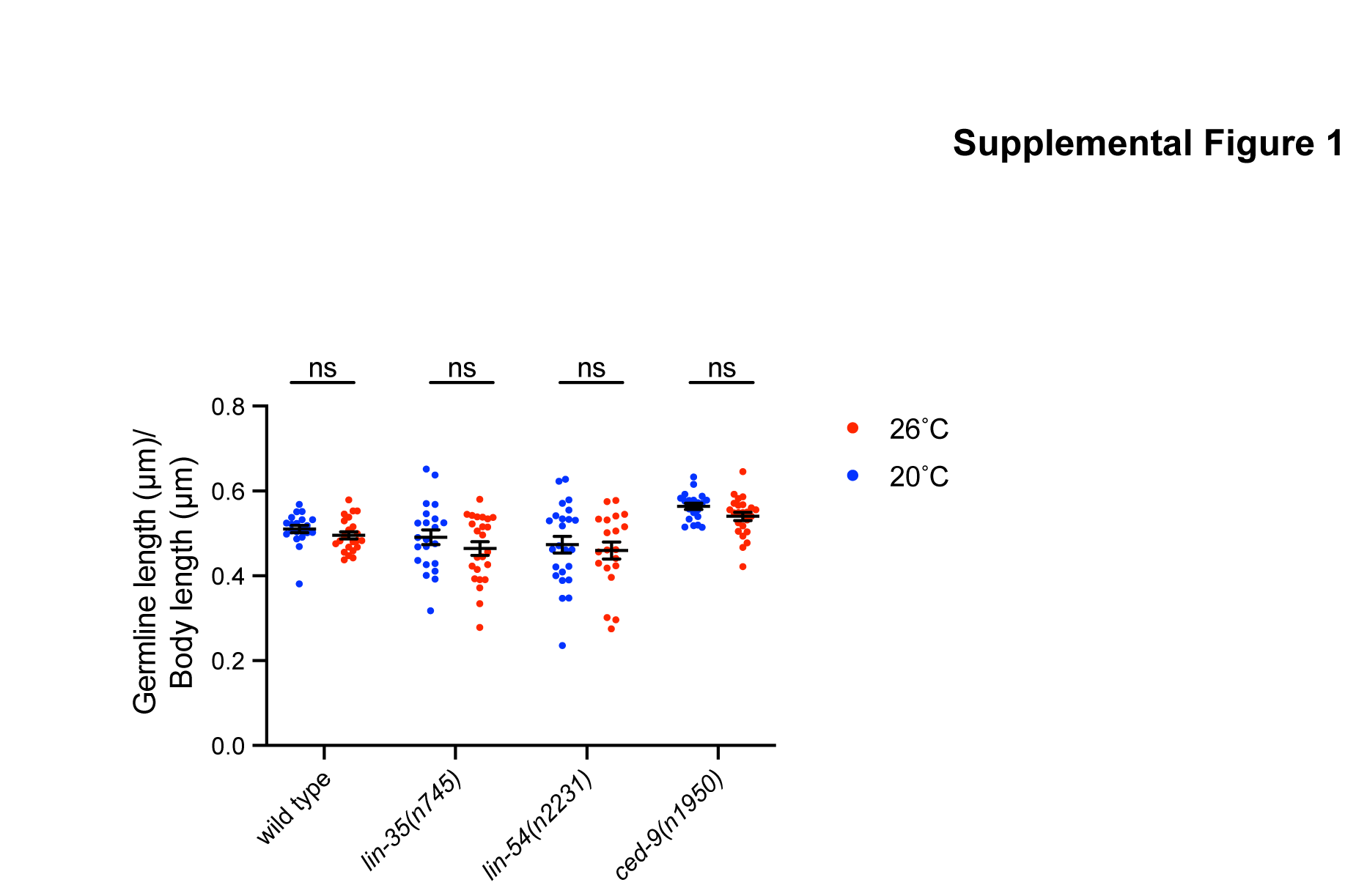

Supplement: jkaf228_Supplementary_Data [file jkaf228_supplementary_data.zip › Supplemental_Figure_1_G3-2025-406020.tif]
